# Editorial: Environmental threats to human reproduction

**DOI:** 10.3389/fendo.2024.1517200

**Published:** 2024-12-02

**Authors:** Roland E. Akhigbe

**Affiliations:** ^1^ Department of Physiology, Ladoke Akintola University of Technology, Ogbomoso, Oyo, Nigeria; ^2^ Reproductive Biology and Toxicology Research Laboratory, Oasis of Grace Hospital, Osogbo, Osun, Nigeria

**Keywords:** environmental toxicant, environmental stressors, oxidative stress, epigenetics, inflammation, apoptosis

The human reproduction involves a cascade of complex events that is controlled by several social, biological, and environmental factors. Environmental factors include heavy metals such as arsenic and lead (1–3), pesticides (4), industrial chemicals like phthalates and bisphenol A (5–7), infection (8), and endocrine disruptors which include pharmaceuticals (9). Environmental toxicants disrupt menstrual cycles, and reduce ovarian reserve and oocyte quality (10, 11). These toxicants also reduce circulating testosterone and sperm quality (12).

More so, these stressors iinfluence pregnancy outcomes. They induce miscarriage and stillbirth, birth defects, preterm birth and low birth weight, and neurodevelopmental disorders (13, 14).

These environmental stressors act via multiple pathways. First, they disrupt the endocrine system by mimicking or blocking sex hormones (15). They may also induce oxidative stress by upregulating the generation of free radicals and suppressing antioxidants (16, 17), trigger inflammation and immune response (18, 19), and promote genotoxicity (20). This Research Topic provides emerging evidences linking environmental toxicants with human reproduction.

Wu et al. reviews the impact of taxanes on ovarian function in women and analyzed the possible reasons for different outcomes. They reported that taxanes-induced ovarian damage is associated with abnormal cell division, follicular cell apoptosis, and reactive oxygen species accumulation. Pan et al. observed an inverted U-shaped association of blood lead levels with oestrogen and a U-shaped association between blood lead levels and sex hormone-binding globulin in female adolescent, indicating that adjusting blood lead exposure to mitigate the effects of lead on growth and development is important for adolescents. He and Wan demonstrated a positive association between smoking and elevated infertility risk.

In a meta-analysis by Hamed et al., it was observed that organophosphate pesticides reduced sperm quality via a testosterone-independent mechanism. Odetayo et al. reported that omega 3 fatty acid attenuated bisphenol F-induced reductions in testosterone and sperm quality by downregulating oxidative stress, inflammation, and apoptosis. Sustarsic et al. observed in a meta-analysis that lifestyle intervention may be beneficial in overweight and obese women diagnosed with infertility by improving ovulation, chances of pregnancy, and rate of live births.

Yao et al. demonstrated a positive association between phthalate exposure and antral follicular count, suggesting that this plasticizer may promote primordial follicle recruitment and depletion of ovarian reserve. Wang et al. demonstrated an inverse association between Life’s simple 7 (LS7) metric scores and infertility. They showed that higher LS7 scores are associated with reduced fertility among women between 18 and 44 years. This finding provides a novel evidence linking cardiovascular status with reproductive health. Qi et al. observed a positive correlation between higher dietary inflammatory index (DII) score and female infertility.

Although SARS-CoV-2 remains quite novel, convincing evidences have been provided on its possible link with infertility (21, 22). Hu et al. showed that asymptomatic or mild SARS-CoV-2 infection during controlled ovarian stimulation had no adverse effect on assisted reproductive technique outcome. Although they observed mild inflammation in the serum, this was absent in the follicular fluid of the subjects. Liprino et al. showed that phase angle is positively associated with low sperm quality. This confirms the reliability of phase angle as a marker of membrane integrity (23). Yu et al. provided a review on the role of epigenetics in female reproduction. They revealed that environmental toxicants impair female reproductive functions via the induction of epigenetic modification.

Summing up, this Research Topic provides interesting data, from experimental to clinical and meta-analysis, demonstrating the influence of environmental stress on human reproduction.

## References

[1] Besong EE Ashonibare PJ Obembe OO Folawiyo MA Adeyemi DH Hamed MA. Zinc protects against lead-induced testicular damage via modulation of steroidogenic and xanthine oxidase/uric acid/caspase 3-mediated apoptotic signaling in male Wistar rats. Aging Male. (2023) 26:2224428. doi: 10.1080/13685538.2023.2224428 37351853

[2] Adeogun AE Ogunleye OD Akhigbe TM Oyedokun PA Adegbola CA Saka WA. Impact of arsenic on male and female reproductive function: a review of the pathophysiology and potential therapeutic strategies. Naunyn-Schmiedeberg’s Arch Pharmacol. (2024), 1–15. doi: 10.1007/s00210-024-03452-6 39287676

[3] Akhigbe RE Akhigbe TM Adegbola CA Oyedokun PA Adesoye OB Adeogun AE. Toxic impacts of arsenic bioaccumulation on urinary arsenic metabolites and semen quality: a systematic and meta-analysis. Ecotoxicology Environ Saf. (2024) 281:116645. doi: 10.1016/j.ecoenv.2024.116645 38941661

[4] Akhigbe RE Oyedokun PA Akhigbe TM Adenike S Oladipo AA Hughes JR. Does pyrethroid exposure lower human semen quality? a systematic review and meta-analysis. Front Toxicol. (2024) 6:1395010. doi: 10.3389/ftox.2024.1395010 38919453 PMC11196980

[5] Castellini C Totaro M Parisi A D’Andrea S Lucente L Cordeschi G. Bisphenol A and male fertility: Myths and realities. Front Endocrinol. (2020) 11:353. doi: 10.3389/fendo.2020.00353 PMC7304337 32595601

[6] Khasin LG Della Rosa J Petersen N Moeller J Kriegsfeld LJ Lishko PV. The impact of di-2-ethylhexyl phthalate on sperm fertility. Front Cell Dev Biol. (2020) 8:426. doi: 10.3389/fcell.2020.00426 32695775 PMC7338605

[7] Pivonello C Muscogiuri G Nardone A Garifalos F Provvisiero DP Verde N. Bisphenol A: an emerging threat to female fertility. Reprod Biol Endocrinol. (2020) 18:1–33. doi: 10.1186/s12958-019-0558-8 32171313 PMC7071611

[8] Ashonibare VJ Ashonibare PJ Akhigbe RE Akhigbe RE. SARS-CoV-2 impairs male fertility by targeting semen quality and testosterone level: A systematic review and meta-analysis. PLoS One. (2024) 19:e0307396. doi: 10.1371/journal.pone.0307396 39250513 PMC11383251

[9] Akhigbe RE Akhigbe TM Oyedokun PA Famurewa AC. Molecular mechanisms underpinning the protection against antiretroviral drug-induced sperm-endocrine aberrations and testicular toxicity: A review. Reprod Toxicol. (2024), 108629. doi: 10.1016/j.reprotox.2024.108629 38825169

[10] Krisher RL. *In vivo* and in *vitro* environmental effects on mammalian oocyte quality. Annu Rev Anim. Biosci. (2013) 1:393–417. doi: 10.1146/annurev-animal-031412-103647 25387025

[11] Ge W Li L Dyce PW De Felici M Shen W. Establishment and depletion of the ovarian reserve: physiology and impact of environmental chemicals. Cell Mol Life Sci. (2019) 76:1729–46. doi: 10.1007/s00018-019-03028-1 PMC11105173 30810760

[12] Kumar N Singh AK. Impact of environmental factors on human semen quality and male fertility: a narrative review. Environ Sci Europe. (2022) 34:1–13. doi: 10.1186/s12302-021-00585-w

[13] Amadi CN Igweze ZN Orisakwe OE. Heavy metals in miscarriages and stillbirths in developing nations. Middle East Fertility Soc J. (2017) 22:91–100. doi: 10.1016/j.mefs.2017.03.003

[14] Beames TG Lipinski RJ. Gene-environment interactions: aligning birth defects research with complex etiology. Development. (2020) 147:dev191064. doi: 10.1242/dev.191064 32680836 PMC7375468

[15] Akhigbe RE Afolabi OA Ajayi AF. L-Arginine reverses maternal and pre-pubertal codeine exposure-induced sexual dysfunction via upregulation of androgen receptor gene and NO/cGMP signaling. PLoS One. (2022) 17:e0274411. doi: 10.1371/journal.pone.0274411 36099318 PMC9469994

[16] Kumar SB Dada R Gupta NP. Environmental toxicants–induced male reproductive toxicity: role of oxidative stress. In: Bioenvironmental Issues Affecting Men’s Reproductive and Sexual Health. Eds: Sikka SC, Hellstrom JG. Academic Press (An imprint of Elsevier, UK) (2018). p. 305–22.

[17] Akhigbe RE Hamed MA Aremu AO. HAART exacerbates testicular damage and impaired spermatogenesis in anti-Koch-treated rats via dysregulation of lactate transport and glutathione content. Reprod Toxicol. (2021) 103:96–107. doi: 10.1016/j.reprotox.2021.06.007 34118364

[18] Ashonibare VJ Akorede BA Ashonibare PJ Akhigbe TM Akhigbe RE. Gut microbiota-gonadal axis: the impact of gut microbiota on reproductive functions. Front Immunol. (2024) 15:1346035. doi: 10.3389/fimmu.2024.1346035 38482009 PMC10933031

[19] Li H Wang XR Hu YF Xiong YW Zhu HL Huang YC. Advances in immunology of male reproductive toxicity induced by common environmental pollutants. Environ Int. (2024) 108898. doi: 10.1016/j.envint.2024.108898 39047547

[20] Choudhuri S Kaur T Jain S Sharma C Asthana S. A review on genotoxicity in connection to infertility and cancer. Chemico-Biological Interact. (2021) 345:109531. doi: 10.1016/j.cbi.2021.109531 34058178

[21] Akhigbe RE Hamed MA. Possible links between COVID-19 and male fertility. Asian Pacific J Reprod. (2020) 9:211–4. doi: 10.4103/2305-0500.294662

[22] Adeyemi DH Odetayo AF Hamed MA Akhigbe RE. Impact of COVID 19 on erectile function. Aging Male. (2022) 25:202–16. doi: 10.1080/13685538.2022.2104833 35924485

[23] Ward LC Brantlov S. Bioimpedance basics and phase angle fundamentals. Rev Endocrine Metab Disord. (2023) 24:381–91. doi: 10.1007/s11154-022-09780-3 PMC10140124 36749540

